# A method to calculate the number of wheat seedlings in the 1st to the 3rd leaf growth stages

**DOI:** 10.1186/s13007-018-0369-5

**Published:** 2018-11-16

**Authors:** Tao Liu, Tianle Yang, Chunyan Li, Rui Li, Wei Wu, Xiaochun Zhong, Chengming Sun, Wenshan Guo

**Affiliations:** 1 Jiangsu Key Laboratory of Crop Genetics and Physiology/Co-Innovation Center for Modern Production Technology of Grain Crops, Yangzhou University, Yangzhou, 225009 China; 2 Key Laboratory of Agro-information Services Technology, Ministry of Agriculture, Beijing, 100081 China

**Keywords:** Wheat seedling numbers, 1st to the 3rd leaf stages, Image processing, Models, Multivariate analysis

## Abstract

**Background:**

The number of cultivated wheat seedlings per unit area allows calculation of plant density. Wheat seedling density provides emergence data and this is useful for improving crop management. The number of wheat seedlings is typically determined by visual counts but this is time-consuming and laborious.

**Results:**

We obtained field digital images of 1st to 3rd leaf stage wheat seedlings. The seedlings were extracted using an image analysis technique that calculated the coverage degree of the seedlings and the number of angular points of overlapping leaves. The wheat seedling quantity estimation model was constructed using multivariate regression analysis. The model parameters included coverage degree, number of angular points, variety coefficient, and leaf age. Introduction of the number of angular points increased the accuracy of the single coverage degree model. The R^2^ value was consistently > 0.95 when the model was applied to different varieties, indicating that the model was adaptable for different varieties. As the leaf stage or density increased, the accuracy of the model declined, but the minimum R^2^ remained > 0.87, indicating good adaptability of the model to seedlings with different leaf ages and densities.

**Conclusions:**

This method is an effective means for counting wheat seedlings in the 1st to the 3rd leaf stages.

## Background

Wheat yield and quality are affected by planting density [1, 2]. An optimal density of wheat seedlings provides the best canopy structure and yield. Determining the number of wheat seedlings per unit area provides information on seedling emergence and is the basis for subsequent cultivation and management [3]. The number of seedlings is usually determined manually but manual count is laborious.

Image analysis techniques have been applied to several aspects of plant production. Common applications include estimation of crop biomass [4], diagnosis of nutritional status [5], analysis of growth [6], monitoring of fertility processes [7], analysis of crop structure [8], and monitoring of diseases, insects, pests, and weeds [9, 10]. Image analysis has also been used to study plant quantitative traits [11, 12], such as fruit counts [13, 14], crop grain counts [15], and pest counts [16]. For the wheat crop, some researchers analyzed the phenotypic traits by image processing technology [17, 18]. There are, however, few reports on the use of image analysis for wheat seedling counts. Recently, researchers have proposed a method for wheat plant counting at the emergence stage based on high spatial resolution RGB images taken from the ground level either from a rover system or from hand held cameras [19]. For reaching the high throughput required for field phenotyping, Jin et al. [20]. proposed a plant counting method from very low altitude unmanned aerial vehicle (UAV) imagery. Although these studies are very useful at the emergence stage of wheat crops, it may be unable to count plants at other leaf stages. Wheat seedlings are not as regular in shape as crop grains or fruits. As a result, overlapping objects are difficult to separate. For the reason of curled leaves, the posture of seedlings are diverse. It also add to the difficulty of segmentation. Finally, there are differences between different varieties of wheat seedlings. Therefore, the image segmentation and counting methods used in previous studies are not useful for counting wheat seedlings.

We optimized the skeleton structure of wheat seedlings using the freeman chain code [21]. Wheat seedlings in the 1st leaf stage were reconstructed into segments to accurately identify each wheat seedling and to complete counting [22]. However, this counting method was restricted to wheat at the 1st leaf stage, the individuals of which can be easily overlooked. Studies on seedling emergence are generally performed during the 1st to the 3rd leaf stages. This provides knowledge of the areas where seedlings are at low density and facilitates additional seeding. In this study, an image analysis technique was used to study methods for rapidly assessing seedling quantity during the 1st–3rd leaf stages, and to analyze differences in the counting of wheat seedlings of different varieties, different leaf ages, and different planting density. We used these data to construct a model for estimating the number of wheat seedlings.

## Methods

### Field experiment and image acquisition

Three wheat varieties, YM23, YF4, and HM7, were manually planted. Two sites in China were studied in 2016: Huaian, Yangzhou. The treatments at sites were the same. The date of sowing was 11 November 2016. The wheat cultivar and experimental design are shown in Table 1.The size of all the plots is 3 m × 4 m, all the treatments were repeated once. The N rate was 240 kg ha^−1^. Here, 50%, 10%, 20%, and 20% total nitrogen treatments were applied at the sowing, tillering, jointing, and booting stages, respectively. As noted in Table 1, three common seedling types were selected for the experiments. Coverage of these wheat varieties with different seedling types is different. Coverage of lax seedlings is more than the half erect seedlings when the numbers of leaves are the same, and coverage of erect seedlings is the minimum among these three seedling types [23]. So, the variety parameters (*Va*) were selected to reduce the impact of the coverage difference among varieties.

**Table 1:** Basic information of the experiments

Cultivars	Seedlings type	Density (10^4^ plants ha^−1^)
YM23	Half erect	75
		150
		225
		300
HM7	Lax	75
		150
		225
		300
YF4	Erect	75
		150
		225
		300

**Fig. 1:**
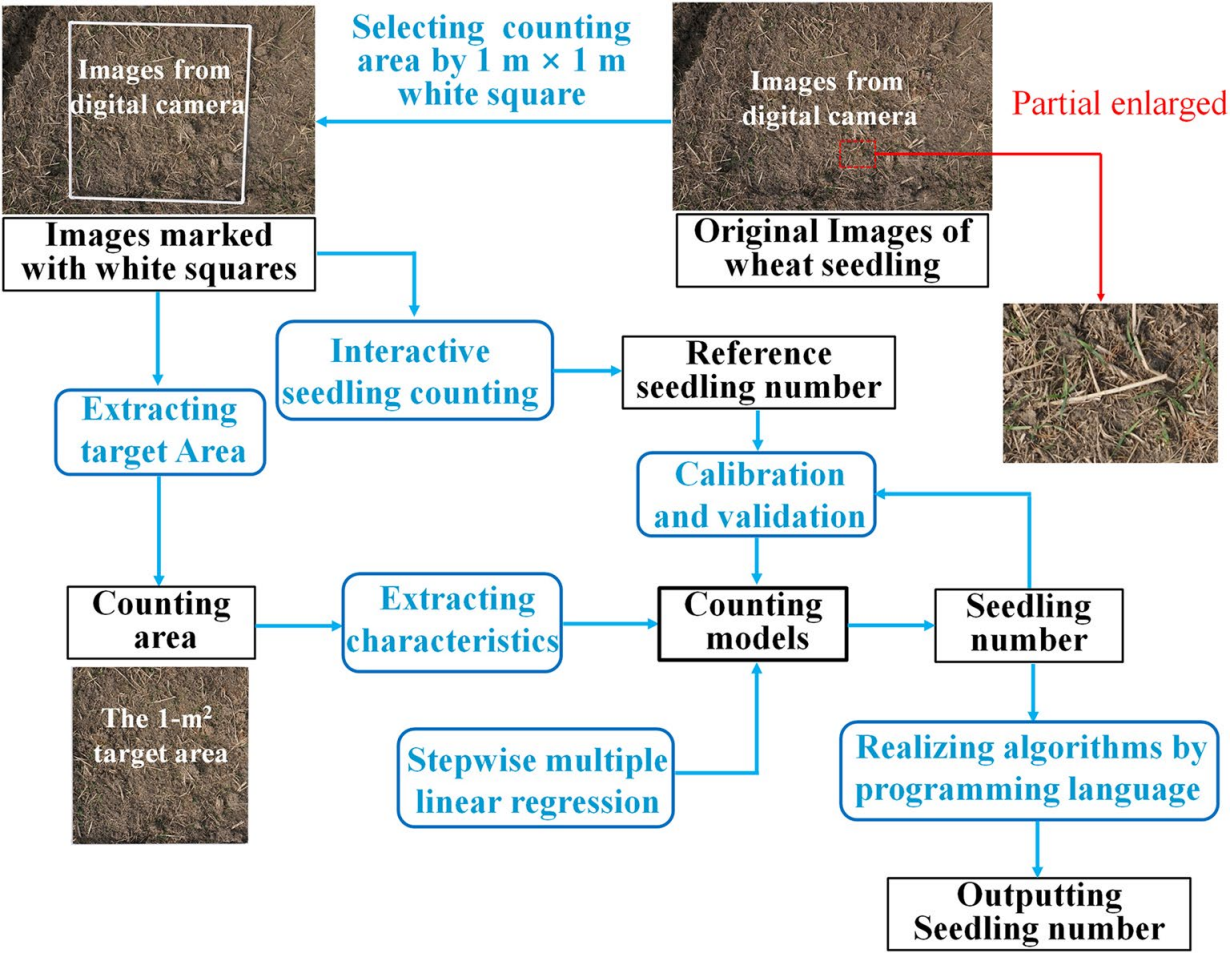
Flowchart of data processing and wheat seedling counting

A Sony Nex5r digital camera was used to capture field images in the late afternoon with dim sunlight or on cloudy days. The camera was set to shutter speed priority with auto adjustment of the ISO up to a maximum of ISO = 1200. The image capture height was approximately 1.5 m, and the counting area was marked by a 1 m × 1 m square. During the 1st–3rd leaf stages, imaging was performed every 3 days and was repeated five times per treatments. The captured images were 6000 × 4000 pixels. Image transformation, cutting, characteristics extracting and regression analysis were made using MATLAB software (version 2015b, MathWorks, USA). Reference seedling numbers were computed from manual visual counts by PhotoShop (version CS6, Adobe Systems Software Ireland Ltd, USA). The flowchart of data processing and wheat seedling counts is shown in Fig. 1.

### Extraction of target area

**Fig. 2:**
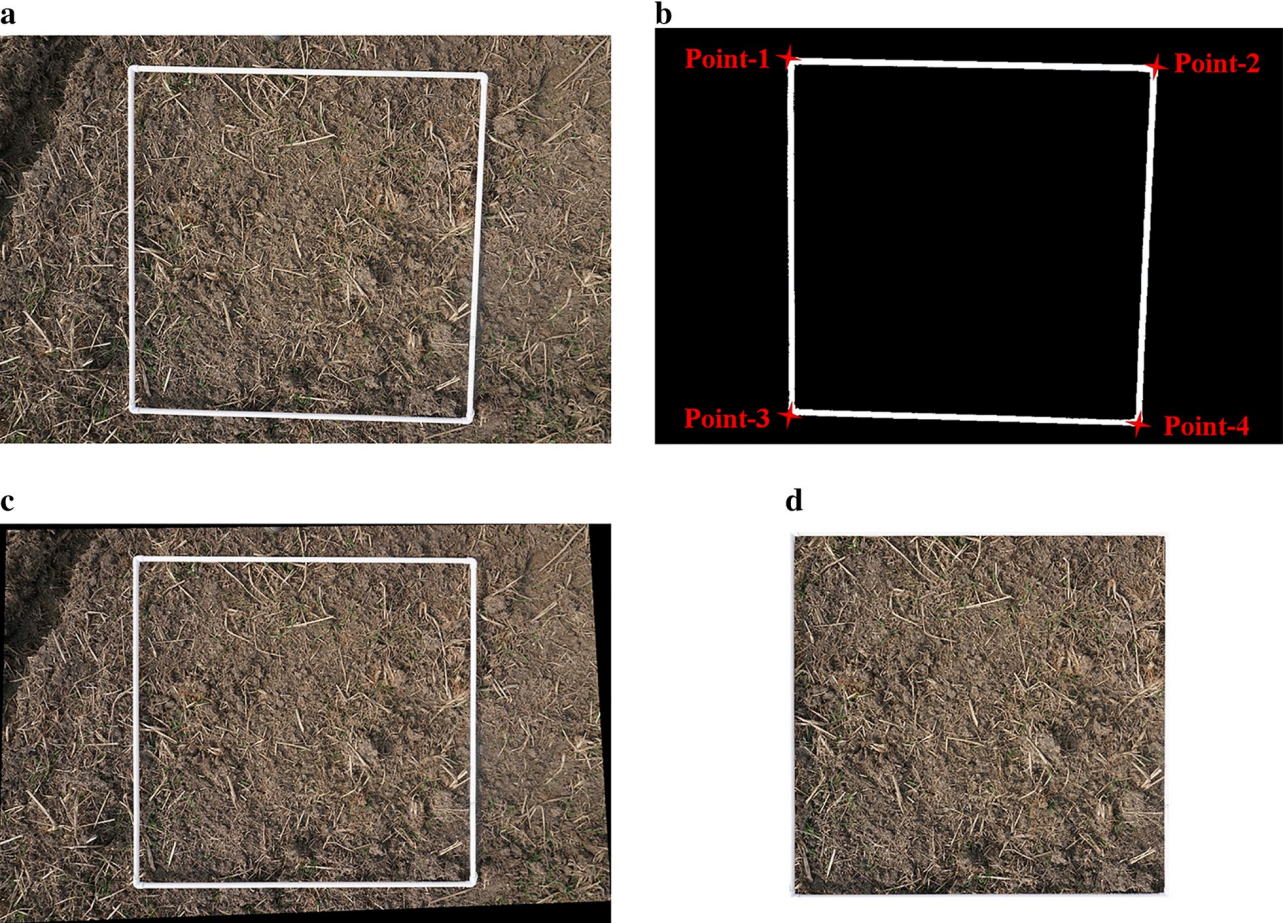
Extraction and rectification of target area: **a** original image; **b** frame extraction and corner detection; **c** perspective transformation; **d** target area extraction

To calculate the number of wheat seedlings, the unit area was extracted first. We selected the area marked by the white quadrangle as the target area (Fig. 2a, which was corrected using the steps below:

1. The white quadrangles in the original images were extracted using Eq. 1. With F(x, y) as the white quadrangle, the color components of red, green, and blue in the RGB images are represented by r, g, and b, respectively. The parameters in Eq. 1 were got by color Features of white quadrangles, wheat seedlings, soil and straws.
  1 $${\text{F}}\left( {{\text{x}},{\text{y}}} \right) = \left\{ {\begin{array}{*{20}l} {r\left( {x,y} \right) + g\left( {x,y} \right) + b\left( {x,y} \right) > 2.1} \hfill \\ {r\left( {x,y} \right) - b\left( {x,y} \right) < 0.05} \hfill \\ \end{array} } \right\}$$
2. Four inflection points of the white quadrangle were extracted. The curvature of the boundary points of the white quadrangle were calculated using Eq. 2, and the 4 angular points were acquired through the variations of the curvature. *C*_(*k*,*i*)_ represents the k neighborhood chain code at the boundary point I; *θi* is the difference of the tangent inclination at the boundary point; and *φ*_*i*_ is the preliminary curvature at the boundary point i. The preliminary curvature value *φ*_*i*_ of the inflection point and its nearby points were relatively large. For this reason, the curvature value *e*_*i*_ represents the inflection point. k is the chain code of the pixel.
  2 $$\left\{ {\begin{array}{*{20}l} {\uptheta_{i} = \left| {C_{{\left( {k, i + \frac{k}{8}} \right)}} - C_{{\left( {k,i} \right)}} } \right|} \hfill \\ {\varphi_{i} = \left\{ {\begin{array}{*{20}l} {\uptheta_{i} } \hfill & {\uptheta_{i} \le k /2} \hfill \\ {k -\uptheta_{i} } \hfill & {\uptheta_{i} > k /2} \hfill \\ \end{array} } \right.} \hfill \\ {e_{i} = \varphi_{i} \mathop \sum \limits_{j = - 1}^{1} \varphi_{i + j} } \hfill \\ \end{array} } \right.$$
3. For the image perspective transformation, the different imaging positions distorted the white-quadrangle area which could influence post-processing. Therefore, a perspective transformation was applied to the images using Eq. 3. The point *u*, *v* was at the right side of the original image. The coordinates after transformation were *x *= *x*′*/w*′, *y *= *y*′*/w*′. The matrix indicates linear transformation and translation. Equation coefficients were solved using the 4 known points.
  3 $$\left[ {x^{\prime } ,y^{\prime } ,w^{\prime } } \right] = \left[ {u,v,w} \right]\left[ {\begin{array}{*{20}c} {a_{11} } & {a_{12} } & {a_{13} } \\ {a_{21} } & {a_{22} } & {a_{23} } \\ {a_{31} } & {a_{32} } & {a_{33} } \\ \end{array} } \right]$$
4. For image cutting, the original white quadrangle was converted to a square in the transformed image. The target area is the inside of the white quadrangle. All the target areas were changed to 800 × 800 pixels.

The extraction results of measurement region are shown in Fig. 2. The white quadrangles in the original images (Fig. 2a) were extracted successfully (Fig. 2b), and four inflection points of the white quadrangle were extracted successfully by the boundary curvature (Fig. 2b). Perspective transformation was solved by 4 known points in Fig. 2b, and the transformative image is shown in Fig. 2c. A 1-m^2^ region was obtained after cutting processing (Fig. 2d). The distortion of the image caused during the photography could be eliminated properly. The results showed that a normative 1-m^2^ region could be obtained accurately by the white squares and image processing methods in Sect. 2.2.

### Extraction of wheat seedlings

Otsu’s method uses a threshold to transform an original image to a foreground and background [24]. In this study, we combined ExG (Eq. 4) and Otsu’s method to extract wheat seedling information. The complete wheat seedlings were extracted by morphologic corrosion, expansion, and hole filling [7]. The MATLAB functions strel, imopen and imfill were used in this step.

4 $$ExG = 1.8 * {\text{g}} - {\text{r}} - {\text{b}}$$

### Extraction of characteristic values

**Fig. 3:**
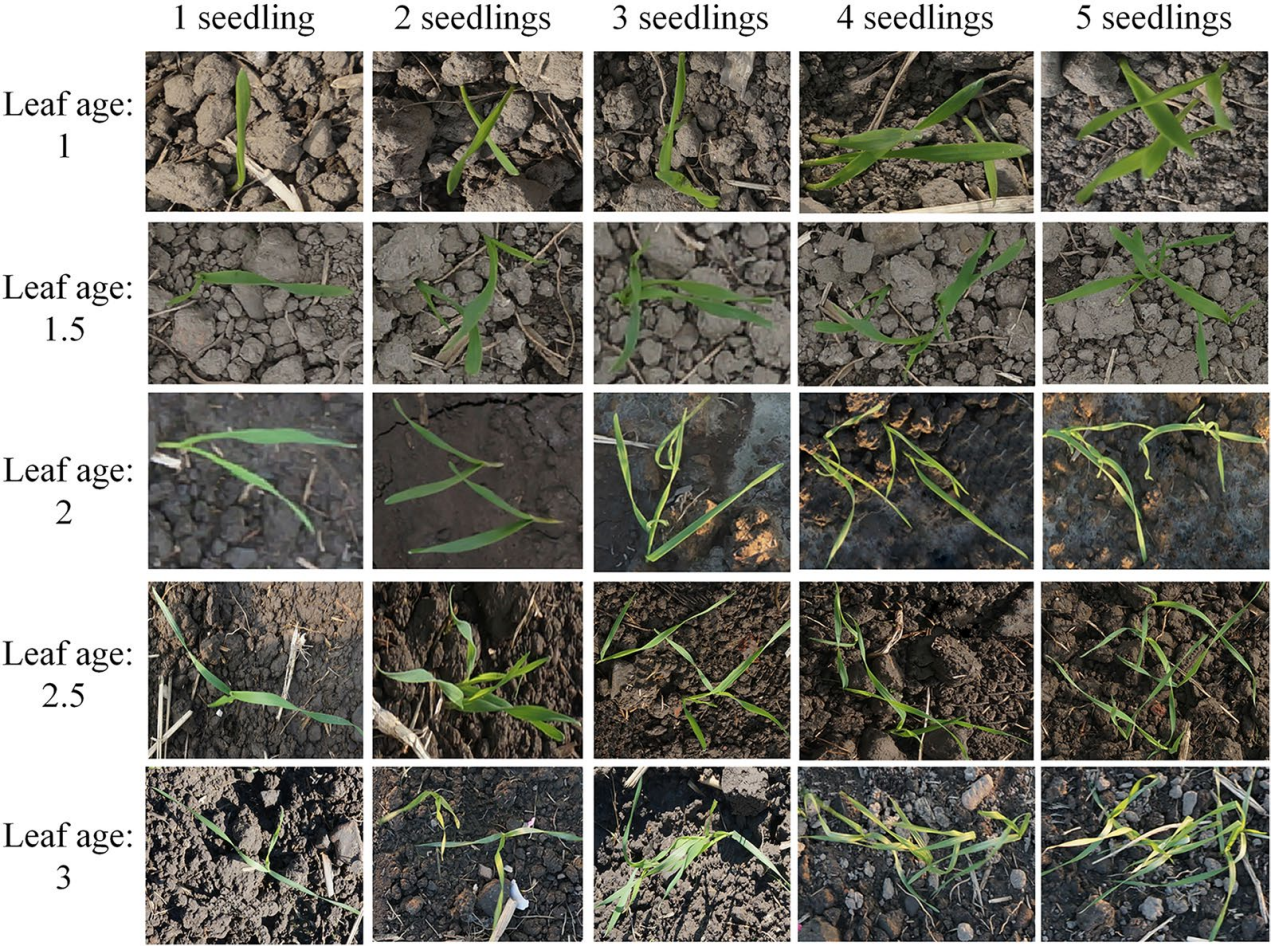
Images of wheat seedlings with different quantities and leaf ages

Images of wheat seedlings with different leaf ages and overlapping situations are shown in Fig. 3. The coverage degree and the number of angular points in the area increased with increased leaf age and the number of overlapping wheat seedlings.

**Fig. 4:**
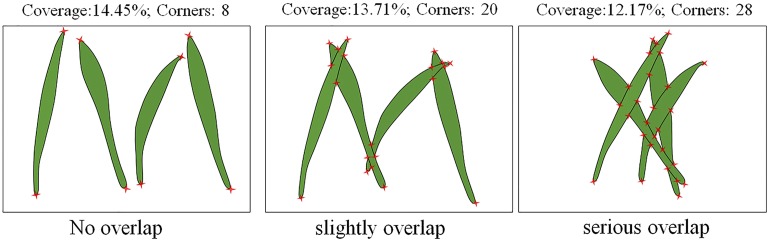
Characteristics of leaves with varying degrees of overlap (only one samples)

**Fig. 5:**
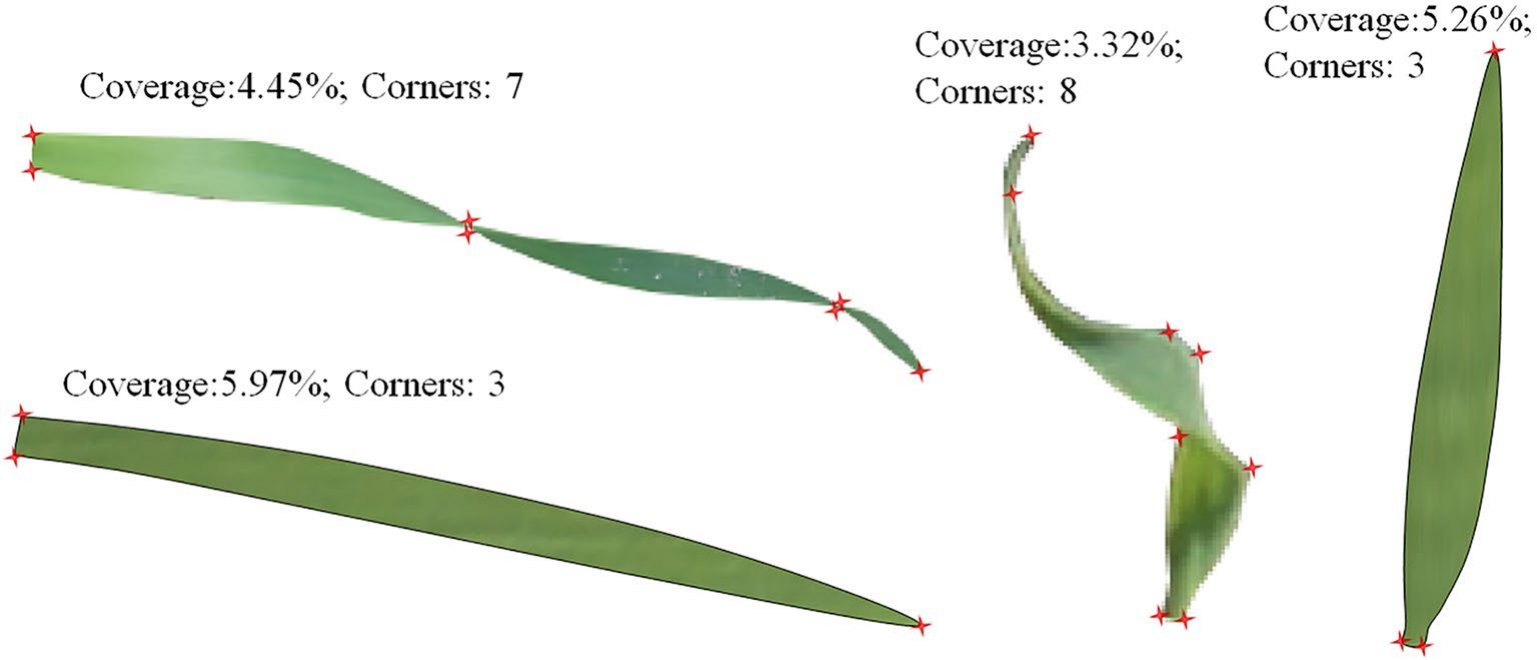
Coverage and corners number of distorted leaves(only one samples)

The greater the density of wheat seedlings in a unit area, the greater the degree of coverage. However, the estimation model is inadequate when constructed using only the coverage degree. The reasons for this are: (1) Coverage degree is affected by overlapped wheat seedlings, and only the reduction of this affect can improve the adaptability of the model. (2) The angular point was the point of the leaf which has a local extremum, it includes leaf tip, the intersection of two leaves (Fig. 4) and distorted leaves (Fig. 5). Angular points will occur on the leaves when the wheat seedlings overlap. The greater the overlap, the more angular points there are. Therefore, the number of angular points can reflect the overlap degree of wheat seedlings. (3) The optimal period to study the number of wheat seedlings is during the 1st–3rd leaf stages. The coverage degree is affected by the difference in leaf age, so the leaf age should be considered when establishing a model. (4) Different varieties of wheat have seedlings with different leaf sizes and this will also influence the degree of coverage. Wheat variety is a key factor in developing an estimation model. For these reasons, we believe that a model that accurately estimates the number of wheat seedlings per unit area should include the following: coverage degree of wheat seedlings (Co), number of angular points (Ha) that reflect the degree of overlap, leaf age (La), and wheat variety (Va). The leaf age and variety were acquired manually.

The coverage degree is the ratio of pixels of leaves to total pixels per unit area. It is calculated by Eq. 5 [6].

5 $$Co = \frac{Seedling Area}{\text{Image area}}$$

The angular points in the images were detected by Eqs. 6–8. E(u, v) is the change value of grayscale; u and v are the movement amount of horizontal and vertical directions; *Ix*, *Iy* is the grayscale value of the image; w(x, y) is the window function; and R is the response function of angular points [25]. When the displacements of the window along x and y direction were *u* and *v*, respectively, the grayscale change can be given by Eqs. (6, 7).

6 $$\begin{aligned} {\rm E}(u,v) & = \sum\limits_{x,y} {w(x,y)} \left[ {{\rm I}\left( {x + u,y + \nu } \right) - {\rm I}\left( {x,y} \right)} \right]^{2} \\ & = [u,\nu ]\sum\limits_{x,y} {w(x,y)} \left[ {\begin{array}{*{20}c} {I_{x}^{2} } & {I_{x} I_{y} } \\ {I_{x} I_{y} } & {I_{y}^{2} } \\ \end{array} } \right]\left[ {\begin{array}{*{20}c} u \\ \nu \\ \end{array} } \right]\,{ = }[u,\nu ]M\left[ {\begin{array}{*{20}c} u \\ \nu \\ \end{array} } \right] \\ \end{aligned}$$

7 $$M\,{ = }\sum\limits_{x,y} {w(x,y)} \left[ {\begin{array}{*{20}c} {I_{x}^{2} } & {I_{x} I_{y} } \\ {I_{x} I_{y} } & {I_{y}^{2} } \\ \end{array} } \right]$$

In the processing of corner points detection, *E*(*u*, *v*) will change significantly how regardless of the (*u*, *v*) do change, these points are corner points. The value of *M* is the determining factor in corner point detection. Hence, the function of corner can be expressed as

8 $${\text{R}} = \det \left( {\text{M}} \right) - {\text{k}}*trace^{2} \left( M \right)$$

where *det*(*M*) is the determinant of *M*, *trace*(*M*) is the trace of *M. k* is constant, and it takes 0.04 here. *k* is and empirical constant, it takes 0.04–0.06. It depends on the size of the image.

### Influence of leaf morphology on coverage degree and the number of angular points

Normally, the accuracy of wheat seedling estimation is not high when only use coverage alone to model. This is mainly due to the overlapped and curled leaves. Leaves in the images are either independent or overlapping. As shown in Fig. 4, when four identical leaves were present in the same area in the form of non-overlapped, slightly overlapped, and highly overlapped, the coverage degree decreased, and the number of angular points increased. For a given number of leaves, the coverage degree and the number of angular points were negatively correlated. Curling of leaf blades affects the degree of coverage. Because of leaf curling, the number of angular points increased while the coverage degree decreased (Fig. 5). Thus, the number of angular points is an important parameter reflecting the overlap of wheat seedlings and the curling of leaves. Combining the number of angular points with coverage degree can improve the accuracy of wheat seedling estimation.

### Model construction

The datasets (images) were separated into two sub-datasets for model training (720 observations in Huaian) and model validation (720 observations in Yangzhou). As the first step for model calibration, correlation analysis was performed between coverage degree (*Co*) and the number of wheat seedlings. The number of angular points (*Ha*) was also increased with the number of wheat seedlings. As the relationship between *Co* and seedlings number was found to be significantly different among leaf stages and varieties, the parameters of leaf age (La) and variety (Va) should be considered to count seedlings number more correctly. Thus, the stepwise multiple linear regression (SMLR) analysis was adopted to calibrate models to count the number of wheat seedlings by using *Co*, *Ha*, La and Va as predictor variables. SMLR method is based on the assumption that linear relationship exists between the number of wheat seedlings and Co. The performance of model equations derived from SMLR analysis was evaluated by the coefficients of determination (R^2^), adjusted R^2^ (A-R^2^), the root mean square error in prediction (RMSE), and the relative error in prediction (REP). R^2^ and RMSE were used to describe the stability of the model and the mean deviation between the measured value and the true value. Adjusted R^2^ and REP were used to evaluate the prediction accuracy of the model. In addition, variety, leaf age, and density were verified.

## Results and analysis

### Extraction of wheat seedlings

**Fig. 6:**
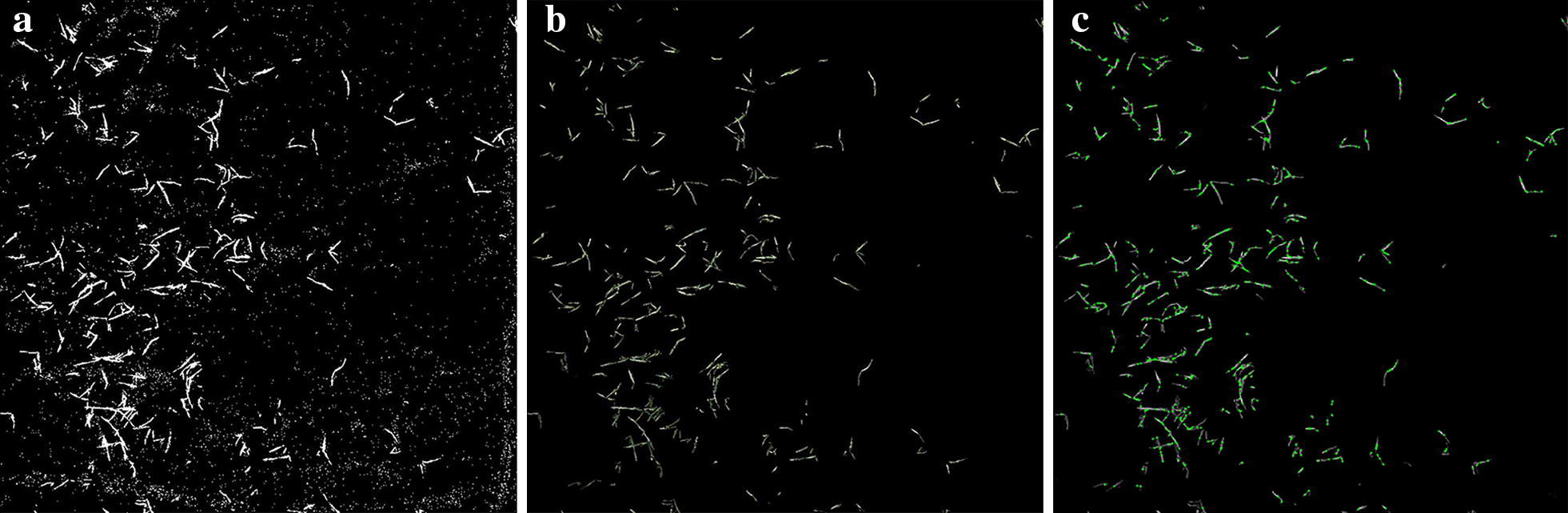
Wheat seedling extraction and angular point detection: **a** segmentation results by ExG; **b** morphological processing results; **c** corner point detection results

The wheat seedlings in the target area were extracted using Eq. 4. Some inclusions (Fig. 6a) were eliminated using morphologic corrosion and expansion (Fig. 6b) to improve the calculation accuracy of the coverage degree and detection of angular points. Figure 6c shows that the inflection point of wheat seedlings in the images can be detected accurately by Eqs. 6–8, providing accurate basic parameters for later model construction. The results suggested that accurate extraction of seedlings should combine color indices and mathematical morphology operation. Further, accurate extraction is the basis for measurement of the coverage degree and detection of angular points.

### Single-factor coverage degree model

**Fig. 7:**
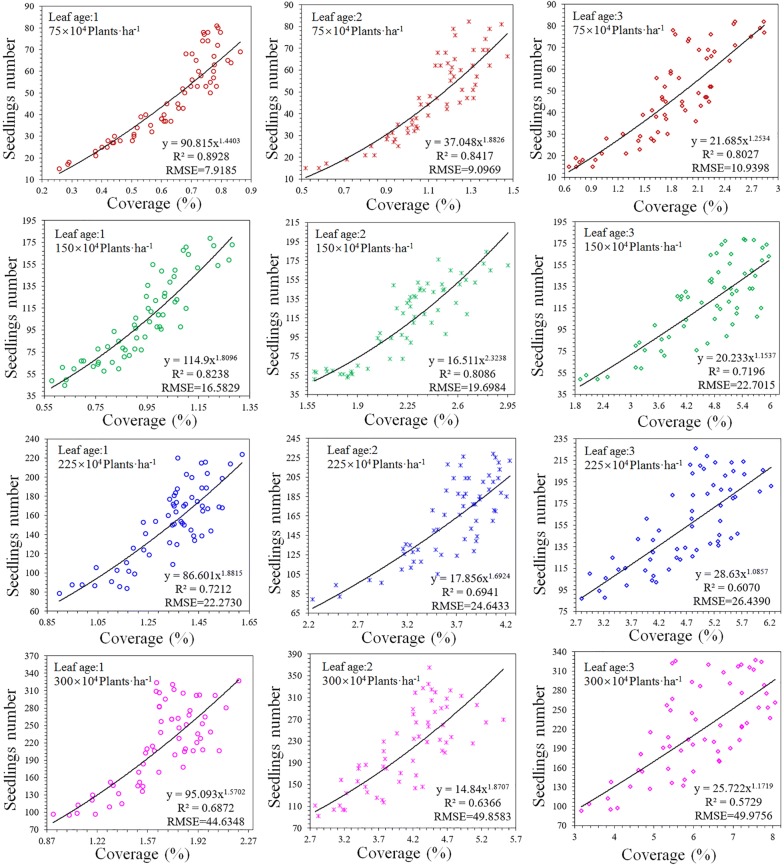
Estimation of the number of wheat seedlings with different leaf ages and densities using the coverage degree. The datasets are these images for model training (720 observations), and each treatment has 60 data. The densities of images from the first line to the fourthly are 75 × 10^4^ plants ha^−1^, 150 × 10^4^ plants ha^−1^, 225 × 10^4^ plants ha^−1^, 300 × 10^4^ plants ha^−1^

The coverage degree of wheat seedlings is highly correlated to their quantity at the 1st–3rd leaf stages. The effect of the wheat seedling estimation model at different periods constructed solely using the coverage degree is shown in Fig. 7. R^2^ of the estimation model reached a peak at the 1st stage, but declined with an increase in density or leaf age. R^2^ ranged from 0.57 to 0.89. Even though the coverage degree can be used to predict trends in the number of wheat seedlings, the accuracy was relatively low, especially during the late growth stage and under conditions of high seedling density. The results indicate that there are large differences among the estimation models at different leaf ages when only the coverage degree is used to construct the model. These differences will complicate applications.

### Multi-factor comprehensive model

There was unsatisfactory accuracy of wheat seedling estimation using coverage alone, especially when planting density was high and after the 1st leaf stage. By combining the angular points (*Ha*) with the coverage degree (*Co*), and using the data of 3 varieties at the 1st, 2nd, and 3rd stages, an estimation model was constructed for wheat seedlings of different varieties and with different leaf ages (*La*) using SMLR (Table 2). Compared with estimation using only *Co*, the introduction of Ha significantly increased the R^2^ value and lowered the RMSE and Rep values. The models in Table 2 could get high R^2^ (more than 0.95) and low RMSE (less than 18) when all the data of sowing densities was pooled together to calibrate and validate the model. The results showed that the proposed angular points could reduce the influences of overlapped leaves and distorted leaves when estimating seedling numbers by the coverage degree. However, the estimation model of different varieties and leaf ages varied, which does not support its application.

**Table 2:** Model equations for estimating seedling number (SN) of different leaf ages using coverage degree and angular points

Varieties	La	Models	R^2^	Adjusted-R^2^	RMSE	Rep (%)
YM23	1	SN_1_ = 0.47 × Ha + 119.37 × Co + 5.65	0.9867	0.9864	10.31	7.72
	2	SN_2_ = 0.22 × Ha + 58.64 × Co + 18.43	0.9781	0.9775	12.16	8.79
	3	SN_3_ = 0.15 × Ha + 37.65 × Co + 14.73	0.9706	0.9698	12.98	8.91
HM7	1	SN_1_ = 0.41 × Ha + 97.13 × Co + 11.37	0.9765	0.9759	11.86	9.22
	2	SN_2_ = 0.19 × Ha + 43.93 × Co + 6.94	0.9611	0.9601	13.13	10.63
	3	SN_3_ = 0.13 × Ha + 31.05 × Co + 17.72	0.9508	0.9495	17.32	12.49
YF4	1	SN_1_ = 0.52 × Ha + 125.21 × Co + 2.71	0.9806	0.9801	9.67	8.12
	2	SN_2_ = 0.25 × Ha + 59.35 × Co + 6.21	0.9713	0.9705	12.59	9.19
	3	SN_3_ = 0.16 × Ha + 39.11 × Co + 11.51	0.9549	0.9537	16.39	11.31

Among the different leaf age models in Table 2, the *Ha* and *Co* coefficients declined with increasing leaf age. To normalize the model, the model transition from the 1st to the 3rd stage was transformed into

9 $${\text{SN}} = \frac{{Va \times \left( {a \times Ha + b \times Co + c} \right)}}{d \times La}.$$

where SN is seedlings number, *Va* is the variety coefficient, *Ha* is the overlap degree of leaves, *La* is leaf age, *a*, *b* and *c* are the coefficient. The value of *La* should be confirmed after investigating the proportion of different leaf stages seedlings, and it’s a non-integer. Based on the wheat seedling data of different varieties and leaf ages, *a *= 0.44, *b *= 110.43, *c *= 3.35, and *d *= 1.11, which were obtained by regression analysis. The modeling and validation results of the different varieties are shown in Table 3. The R^2^ value was always > 0.95, and RMSE remained in a small range in both the modeling process and validation process. Adjustment of the variety parameter *Va* made the difference insignificant when the model was applied to different varieties. Compared with separate modeling, the accuracy of the overall model declined slightly, but its application scope and period increased.

**Table 3:** Calibration and validation results of the seedling number estimation model (Eq. 9)

Varieties	*Va*	Training				Validation			
		R^2^	A-R^2^	RMSE	Rep (%)	R^2^	A-R^2^	RMSE	Rep (%)
YM23	1.05	0.9626	0.9620	18.4	8.17	0.9386	0.9370	20.27	9.72
HM7	0.86	0.9533	0.9525	21.84	12.31	0.9183	0.9162	22.23	12.79
YF4	1.12	0.9537	0.9529	23.06	14.25	0.9129	0.9106	22.99	15.01

### Validation of leaf ages and densities

**Fig. 8:**
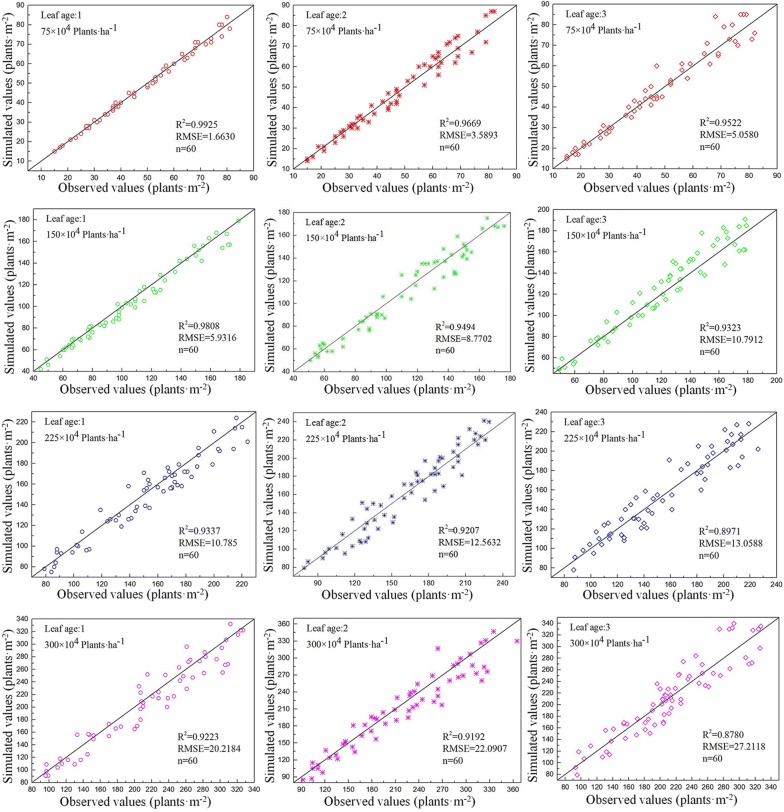
Validation results of seedling counting model of different densities and leaf ages. The datasets are these images for model validation (720 observations), and each treatment has 60 data. The densities of images from the first line to the fourthly are 75 × 10^4^ plants ha^−1^, 150 × 10^4^ plants ha^−1^, 225 × 10^4^ plants ha^−1^, 300 × 10^4^ plants ha^−1^

There are differences in the model application, with respect to seedlings, at different densities and leaf ages. The accuracy of low density was higher than that of high density, and the R^2^ of 75 × 10^4^ density was always > 0.95, while the R^2^ of 300 × 10^4^ density was approximately 0.9 (Fig. 8). The accuracy in the 1st leaf stage was the highest, and its mean value was > 0.95. Accuracy was lowest in the 3rd leaf stage, with a mean R^2^ value of 0.91, and the R^2^ values of 225 × 10^4^ and 300 × 10^4^ were < 0.9. The above R^2^ values were very high and the value of RMSE was small, indicating the reliability and high precision of the results. These results indicate that it is best to get images at the 1st or 2nd leaf stages, especially when plant densities are high.

## Discussion

A multivariate model consisting of coverage degree, angular points, leaf age and variety coefficients was used to estimate the quantity of wheat seedling (Eq. 9). The coverage degree was selected as the main parameter of this model because it is highly correlated with the number of wheat seedlings. The changes in the quantity can be reflected to some extent by solely using coverage degree, which is similar to previous results where coverage degree was used to estimate agronomic parameters [6, 26]. However, the overlap and degree of leaf curl were not considered in those studies. In the present study, the number of angular points strongly reflected the overlap and degree of leaf curl thereby improving the accuracy of estimation compared to a system that solely uses the coverage degree.

**Fig. 9:**
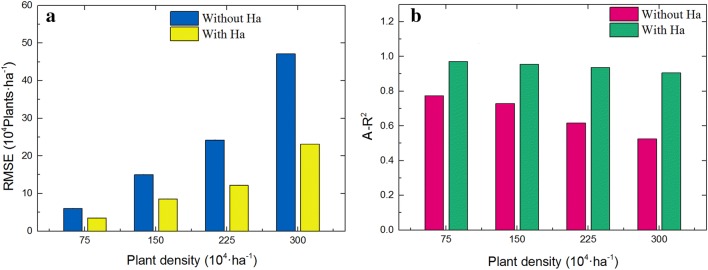
The effect of angular points on improving the accuracy of different plant density: **a** the effect of angular points on RMSE, **b** the effect of angular points on A-R^2^

Plant density is the most important factor that affects the accuracy rate of seedling counting. Our results show that the errors increase with the plant density because of the increased overlap between leaves. This result is consistent with a previous study [19]. However, the angular points (*Ha*) proposed in this study attenuate the increase in errors caused by plant density. As presented in Fig. 9, the effect of Ha on improving the accuracy increases with plant density. Although the accuracy of the proposed model decreased slightly with plant density, a low RMSE was maintained to ensure the reliability of the estimation results.

**Fig. 10:**
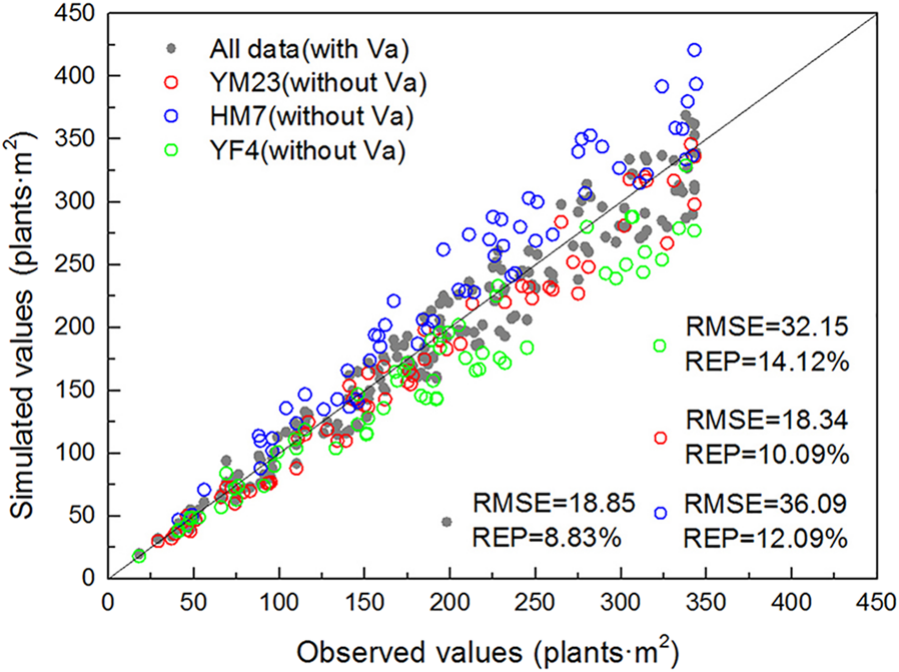
Validation results of the model without variety parameter Va

Three wheat varieties were selected for this study and the leaf shapes of these differed from the 1st to the 3rd stages. The differences between varieties were in leaf size and degree of curl [27, 28]. High accuracy was obtained using the coverage degree and angular points to estimate the number of wheat seedlings of different varieties and leaf ages, respectively (Table 2). However, this led to an excessive number of models and a lack of applicability. Therefore, the variety coefficient Va was introduced into the model, increasing its adaptability for different varieties. As in Fig. 10, the RMSE and REP between the measured and estimated values of the 3 varieties increased when Va was not introduced, especially for HM7 and YF4. Thus, the variety should be considered to improve model accuracy. We determined the Va value of 3 wheat varieties. To apply the model to other varieties, the specific Va value should be determined and used.

**Fig. 11:**
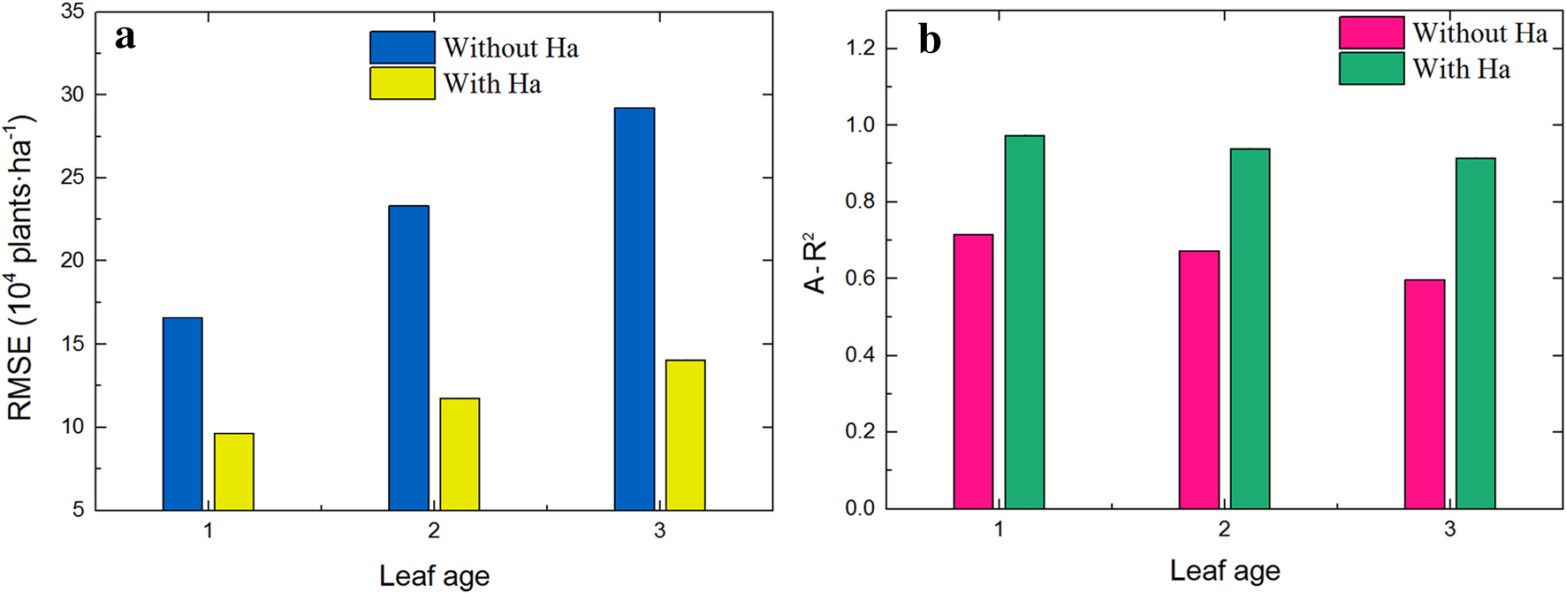
The effect of angular points on improving the accuracy of different leaf ages: **a** the effect of angular points on RMSE, **b** the effect of angular points on A-R^2^

The leaf age at image acquisition is critical for obtaining an accurate estimation of seedling numbers [19, 29]. In previous research, each wheat seedling was reconstructed into a separate line segment by optimizing the 1st-stage seedling structure using the chain code, thus accurately estimating the number of wheat seedlings [22]. In the application, however, it was discovered that the 1st stage persisted only a short time and was easy to miss. The accuracy rate was only 42.36% when the previous algorithm was used to count seedlings numbers at 2nd leaf stage, and 32.12% at 3rd leaf stage. The 1st–3rd leaf stages, which usually last for a total of 25 d, are a useful period to study seedling emergence in the field. There is ample time for follow-up operations, such as seedling supplements, to be performed. The proposed angular points (Ha) greatly reduce the error. Figure 11 shows that the RMSE decreases and R^2^ increases consistently when Ha is used to count seedlings. The effect of Ha on improving the accuracy increases with leaf age. Although the accuracy of the proposed model decreased slightly with leaf age, a low RMSE could be maintained to ensure the reliability of the estimation results. Thus, the model can be applied to the quantity estimation of wheat seedlings of different varieties from the 1st to the 3rd leaf stages.

**Fig. 12:**
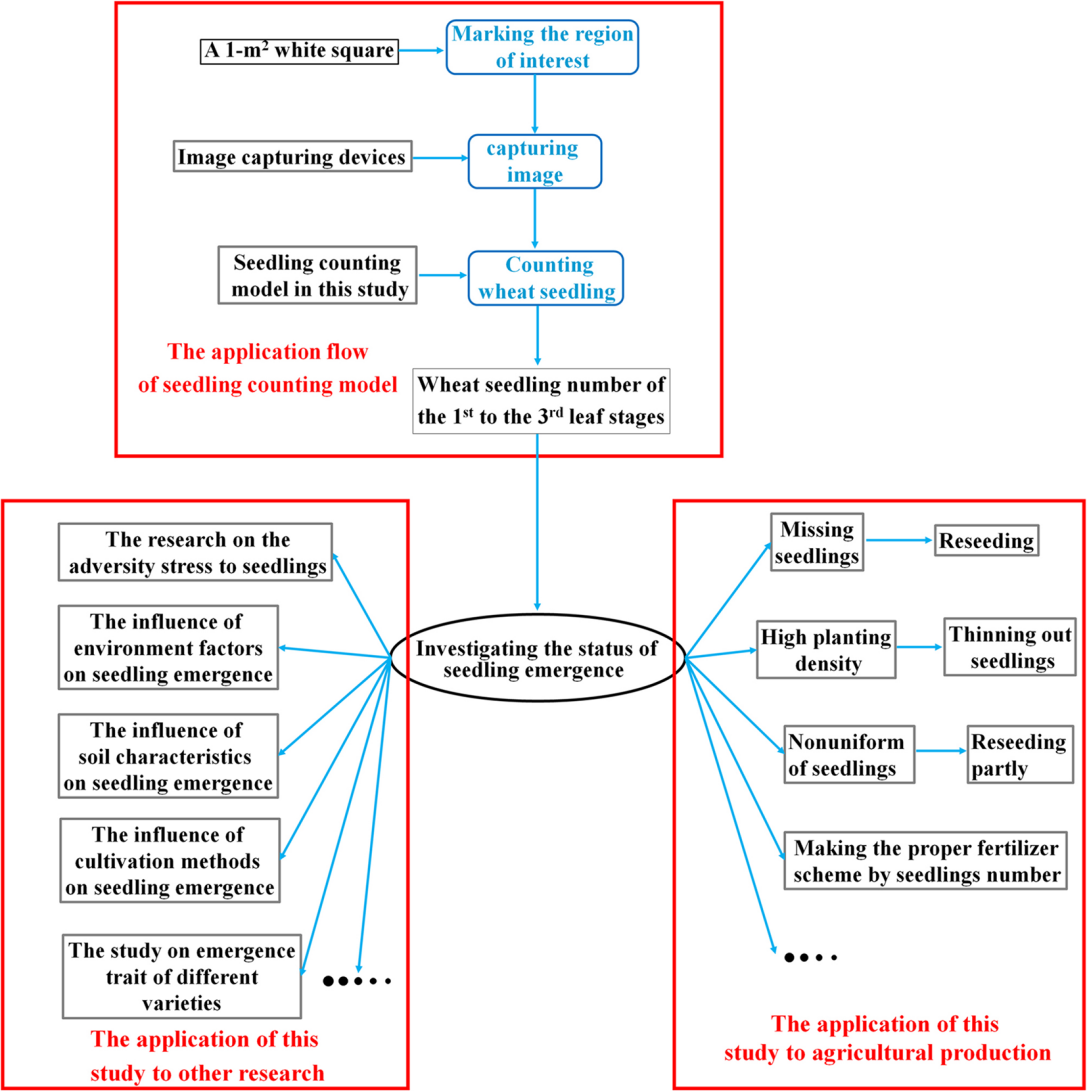
Application flow and the seedling counting method proposed in this study

Seedling number (plant density) is a key factor influencing wheat production with effects on yield, water and fertilizer requirements as well as susceptibility to environmental change [20]. Wheat researchers can obtain an accurate estimate of seedling number using the proposed method and they can then study the influence of external factors or varietal characteristics on seedling emergence (Fig. 12). Wheat producers can investigate the status of seedling emergence using the proposed method and then make necessary adjustments in their crop management schemes (Fig. 12). The application flow of the proposed method is shown in Fig. 12. A 1-m^2^ white square and an image capturing device (digital camera, smart phone, and surveillance camera are all practicable) can be used when original images are needed.

## Conclusion

A wheat seedling estimation model was constructed based on coverage degree and angular points. Its application scope was expanded, and the application period was prolonged using a variety coefficient and leaf age coefficient. The new model explained the unsatisfactory accuracy of a previous estimation model that only used degree of coverage. The new model was improved by the introduction of angular points. The new model could be used to estimate the quantity of wheat seedlings in the 1st to the 3rd stages, and it provides a basis for timely seedling supplements and subsequent crop management.
